# *MtBZR1* Plays an Important Role in Nodule Development in *Medicago truncatula*

**DOI:** 10.3390/ijms20122941

**Published:** 2019-06-16

**Authors:** Can Cui, Hongfeng Wang, Limei Hong, Yiteng Xu, Yang Zhao, Chuanen Zhou

**Affiliations:** 1 The Key Laboratory of Plant Development and Environmental Adaptation Biology, Ministry of Education, School of Life Science, Shandong University, Qingdao 266237, China; zndfq_cuican@outlook.com (C.C.); honglimei227@163.com (L.H.); xuyi.teng@163.com (Y.X.); 2 School of Life Science, Guangzhou University, Guangzhou 510006, China; 654187588@163.com

**Keywords:** MtBZR1, brassinosteroid, nodulation, transcription factor, *Medicago truncatula*

## Abstract

Brassinosteroid (BR) is an essential hormone in plant growth and development. The BR signaling pathway was extensively studied, in which *BRASSINAZOLE RESISTANT 1* (*BZR1*) functions as a key regulator. Here, we carried out a functional study of the homolog of *BZR1* in *Medicago truncatula* R108, whose expression was induced in nodules upon *Sinorhizobium meliloti* 1021 inoculation. We identified a loss-of-function mutant *mtbzr1-1* and generated *35S:MtBZR1* transgenic lines for further analysis at the genetic level. Both the mutant and the overexpression lines of *MtBZR1* showed no obvious phenotypic changes under normal growth conditions. After *S. meliloti* 1021 inoculation, however, the shoot and root dry mass was reduced in *mtbzr1-1* compared with the wild type, caused by partially impaired nodule development. The transcriptomic analysis identified 1319 differentially expressed genes in *mtbzr1-1* compared with wild type, many of which are involved in nodule development and secondary metabolite biosynthesis. Our results demonstrate the role of *MtBZR1* in nodule development in *M. truncatula,* shedding light on the potential role of BR in legume–rhizobium symbiosis.

## 1. Introduction

*Medicago truncatula* is a model fabacean species for nodulation and nitrogen fixation. During the initiation of symbiotic nodules, for example, the root nodule microsymbionts (collectively termed rhizobia) secrete nodulation (Nod) factors, which are lipochito-oligosaccharides functioning as signal molecules, in response to flavonoids from the host plant. The Nod factors are then perceived by the corresponding receptor kinases in the root tissue, which in turn trigger nodule organogenesis and root hair deformation [1]. After nodule formation, the host plant generates a class of nodule-specific cysteine-rich (NCR) peptides to induce the differentiation of endosymbionts into enlarged and polyploid bacteroids [2]. There are about 600 *MtNCR* genes in the *M. truncatula* genome, indicating their important roles in nitrogen fixation [3]. Hormones also play important roles during nodule development [4,5]. 

Flavonoids are known as secondary metabolites that play important roles in legume–rhizobium symbiosis [6]. Based on the molecular structure, flavonoids consist of flavones, flavonols, isoflavones, flavanones, flavanols, and anthocyanins [7]. The flavonoid biosynthesis pathway starts from downstream of the phenylalanine pathway, in which *p*-coumaroyl co-enzyme A (CoA) is produced and catalyzed into chalcone by chalcone synthase. In the flavonoid biosynthesis pathway, chalcone is catalyzed into dihydrokaempferol by chalcone isomerase and flavanone 3-hydroxylase, and then by flavonol synthases into flavonols. Flavonoids production is suggested to be highly localized at infection sites in legume [8]. 

Brassinosteroid (BR) is an essential plant hormone in plant growth and developmental processes, such as cell elongation, photomorphogenesis, seed germination, stomata differentiation and movement, flowering, and plant response to biotic and abiotic stresses [9,10,11]. *AtBZR1* (*Arabidopsis thaliana BRASSINAZOLE-RESISTANT 1*) is a key transcriptional regulator in the BR signaling pathway [12,13,14]. *AtBZR1* has a homolog, *AtBES1* (*At BRASSINOSTEROID INSENSITIVE 1-EMS SUPPRESSOR 1*), which shares 88% similarity in the *A. thaliana* genome [15]. Because of the redundancy with *AtBES1*, the *atbzr1* mutant showed no obvious phenotypic change, while the gain-of-function mutant *atbzr1-D* showed enhanced BR signaling in *A. thaliana*. Recently, *mtbri1* was reported to show defective nodule development, indicating that BR signaling is involved in legume nodulation [16]. As *MtBZR1* is an important transcription factor downstream of *MtBRI1*, it is intriguing to explore its role in nodule development in *M. truncatula*.

This study was undertaken with the aim to determine the function of *MtBZR1* in *M. truncatula* R108 nodule development. In order to achieve this, we isolated mutants of *MtBZR1* and characterized their phenotypes. Next, we performed transcriptomic analysis of the *mtbzr1-1* null mutant, in comparison to the wild-type plants, to identify the deregulated genes. The results indicate that *MtBZR1* is involved in the control of nodule development, but not its initiation, in *M. truncatula*. *MtBZR1* was induced by the rhizobium *Sinorhizobium meliloti* 1021, and *mtbzr1-1* displayed partially impaired nodule development compared to the control R108. In addition, transcriptomic analysis found that the genes involved in nodule development and secondary metabolite biosynthesis were under transcriptional reprogramming, consistent with the morphological changes in *mtbzr1-1*. These results indicate that *MtBZR1* plays a role in controlling nodule development after the initiation in *M. truncatula*.

## 2. Results

### 2.1. Search for *AtBZR1* Homologs in *M. truncatula*

**Figure 1:**
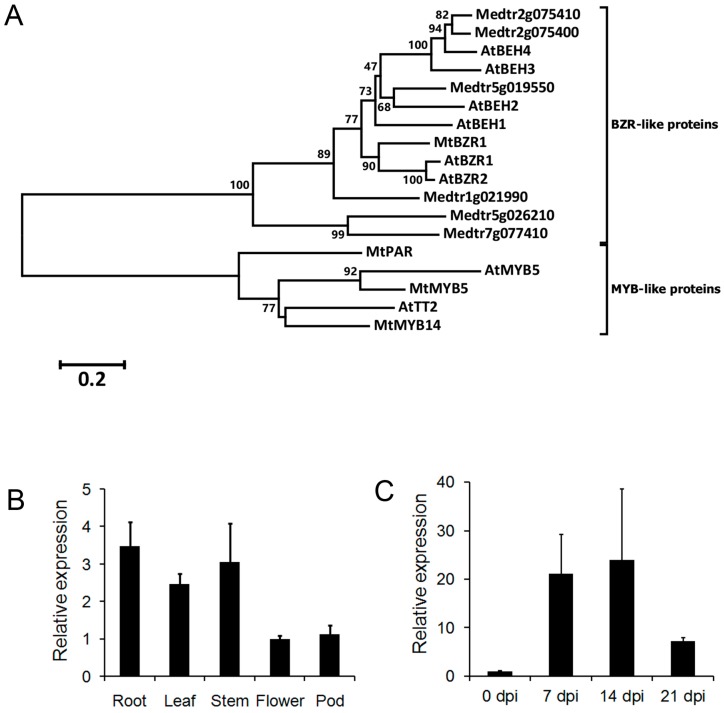
The phylogenetic analysis and expression of *Medicago truncatula brassinazole resistant 1* (*MtBZR1*). (**A**) A neighbor-joining tree of BRASSINAZOLE-RESISTANT 1 (BZR1)-like proteins in *A. thaliana* and *M. truncatula*. AtMYB5 and *A. thaliana* TRANSPARENT TESTA 2 (AtTT2) acted as an outgroup sequence. Multiple alignment and phylogenetic analysis were run with the MEGA6 program with 1000 bootstraps. Scores higher than 50 are labeled by the branches. The protein sequences from *A. thaliana* start with “At”; those from *M. truncatula* start with “Mt”. (**B**) Expression of *MtBZR1* in *M. truncatula* tissues. The expression in the flower was set as 1.0. (**C**) Expression of *MtBZR1* in nodules after inoculation with rhizobium (dpi, days post inoculation). The expression at zero dpi was set as 1.0. In (**B**,**C**), *MtUbiquitin* gene was used as an internal control. Bars represent standard errors from three biological replicates.

We searched the annotated *M. truncatula* genome for the homolog of *A. thaliana AtBZR1*. Medtr3g111680 encodes a 315-amino-acid (aa) protein that shares 64% identify with the *A. thaliana* AtBZR1 protein. According to the phylogenic analysis of 11 *A. thaliana* and *M. truncatula* homologs, Medtr3g111680 is the closest homolog of AtBZR1 in *M. truncatula* (Figure 1A; Figure S1, Supplementary Materials). It is clustered with two other proteins, Medtr1g021990 and AtBZR2. In addition to the high similarity in the protein sequence, Medtr3g111680 also possesses a BZR domain with conservative sites (Figure S2, Supplementary Materials). We, therefore, denoted *Medtr3g111680* as *MtBZR1*. 

### 2.2. Induction of *MtBZR1* upon *S. meliloti* 1021 Inoculation 

To characterize its role in plant development, an expression assay of *MtBZR1* by quantitative real-time PCR (qRT-PCR) was carried out in various tissue types. As shown in Figure 1B, *MtBZR1* expression was highest in the root tissue, and comparable in stem and leaf, and relatively low in flower and pod. As nodulation is a specific characteristic of legume, we then performed another expression assay in nodules of the *M. truncatula* R108. As shown in Figure 1C, *MtBZR1* was induced more than 20-fold in nodules at seven and 14 days post inoculation (dpi) by *S. meliloti* 1021 strain, and then reduced to seven-fold at 21 dpi, compared with that at zero dpi in R108. These results suggest that *MtBZR1* may be involved in nodule development in *M. truncatula*.

### 2.3. Identification of the *mtbzr1-1* Null Mutant

**Figure 2:**
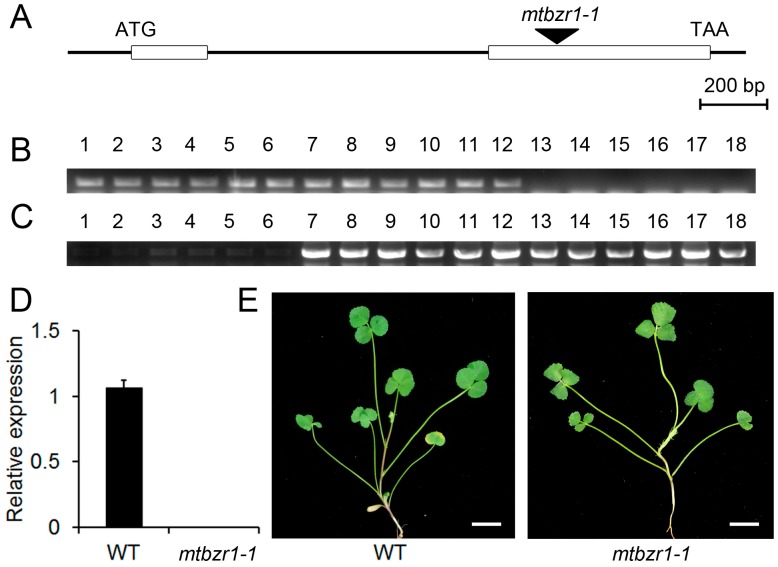
The gene structure of *MtBZR1* and identification of the *mtbzr1-1* mutant. (**A**) The gene structure of *MtBZR1*. The open boxes represent two exons and the *Tnt1* insertion in the null mutant *mtbzr1-1* is indicated by the arrow. (**B**) The PCR of the primers crosses the *Tnt1* insertion bygene-specific primers. Lane 1–6, wild type (WT); lane 7–12, heterozygous mutants; lane 13–18 homozygous mutants. (**C**) PCR using a gene-specific primer and a *Tnt1* primer. (**D**) The expression of *MtBZR1* in the wild type (WT) and *mtbzr1-1*. (**E**) The shoots of one-month-old WT and *mtbzr1-1* plants. Scale bar = 1 cm. In (**D**), *MtUbiquitin* gene was used as an internal control. Bars represent standard errors from three biological replicates.

To illustrate the biological function of *MtBZR1*, we adopted a reverse genetic approach by searching the Noble Research Institute Mutant collection for the loss-of-function mutants. One mutant line (*mtbzr1-1*) that contains a single insertion in *MtBZR1′*s second exon was found and used for the following genetic analysis (Figure 2A). The insertion was confirmed by genotyping using the gene-specific primers and a *Tnt1* primer (Figure 2B,C), and homozygous plants were identified. As shown in Figure 2D, we detected no expression of *MtBZR1* in the *mtbzr1-1* mutant plant. There was no obvious difference in developmental phenotypes between *mtbzr1-1* and wild-type plants (Figure 2E). 

### 2.4. Loss of Function in *MtBZR1* Leads to a Decrease in Plant Dry Mass in *S. meliloti* 1021 Nodulation

**Figure 3:**
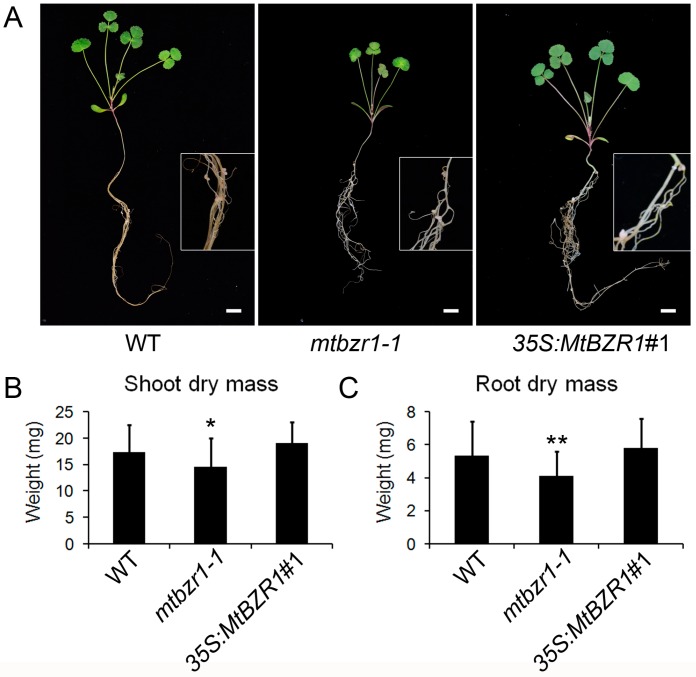
Reduced dry mass in *mtbzr1-1* in nodulation. (**A**) Photos of one-month-old wild-type (WT), *mtbzr1-1*, and *35S:MtBZR1#1* transgenic line. Scale bar = 1 cm. Insets are close views of the roots with nodules in WT, *mtbzr1-1*, and *35S:MtBZR1*#1 transgenic plants. Dry mass of shoot (**B**) and root (**C**) of WT, *mtbzr1-1*, *35S:MtBZR1*#1 transgenic line 21 days after rhizobial inoculation. Values are the means ± SD (*n* = 25); *T* = 2; * *p* < 0.05, ** *p* < 0.01.

The induction of *MtBZR1* in nodulation implies its role in nodule development. Thus, we compared the growth between *mtbzr1-1* mutant and wild type after *S. meliloti* 1021 inoculation. As shown in Figure 3A, the inoculated *mtbzr1-1* mutant exhibited a smaller plant size compared with the wild-type R108. To confirm this observation, we measured the dry mass of the shoot and root tissues in *mtbzr1-1* and wild-type R108 plants 21 days after inoculation. The dry mass of both shoot and root in *mtbzr1-1* was significantly decreased, compared with that of the wild-type R108 control (Figure 3B,C). 

**Figure 4:**
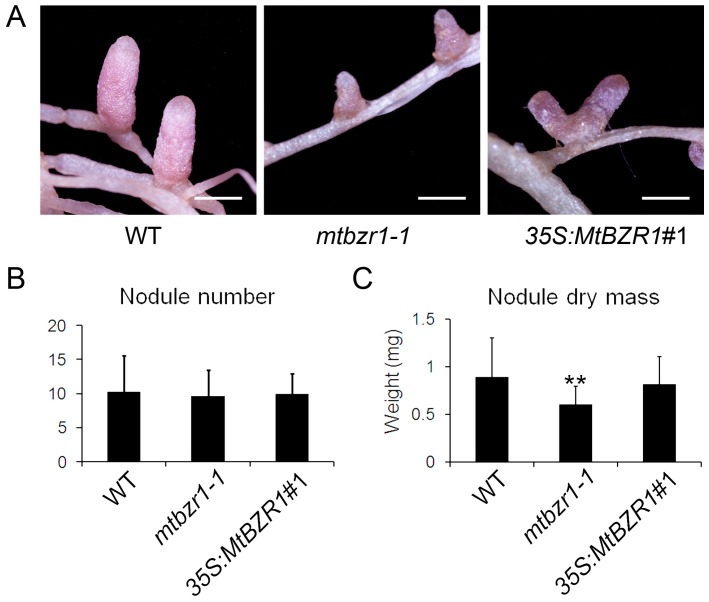
Nodule development in wild type (WT), *mtbzr1-1*, and *35S:MtBZR1*#1 transgenic line. (**A**) Photos of nodules from one-month-old WT, *mtbzr1-1*, and *35S:MtBZR1*#1 transgenic line. Scale bar = 1 mm. Nodule number (**B**) and dry mass (**C**) in WT, *mtbzr1-1*, and *35S:MtBZR1*#1 transgenic line 21 days after rhizobial inoculation. Values are the means ± SD (*n* = 25); *T* = 2; ** *p* < 0.01.

To explore the reason why *mtbzr1-1* growth was retarded, we compared the nodule development in the mutant with the wild-type plants. As shown in Figure 4A, the nodules on *mtbzr1-1* roots were smaller than those from the wild type. The measurement of nodule dry mass confirmed the observation that the nodules from *mtbzr1-1* were significantly smaller/lighter than those from the wild type, although the number of nodules was comparable between them (Figure 4B,C). The genetic assay of *mtbzr1-1* demonstrates that the loss of function in *MtBZR1* leads to partially impaired nodule development in *M. truncatula*.

To further investigate the role of *MtBZR1*, the full length of the *MtBZR1* coding sequence under control of the CaMV 35S promoter was introduced into the wild-type background. A total of six independent transgenic lines were generated and two lines with the highest *MtBZR1* expression were used for further analysis (Figure S3A, Supplementary Materials). The *35S:MtBZR1* plants showed no obvious phenotypic changes with normal compound leaves from wild-type plants (Figure S3B, Supplementary Materials). The recombinant protein fusioned with a green fluorescence protein (GFP) showed the detection of the GFP signal of MtBZR1-GFP (Figure S3C, Supplementary Materials).

When we carried out rhizobial inoculation of the *S. meliloti* 1021 strain on the *35S:MtBZR1* lines, as shown in Figure 3A, the *35S:MtBZR1*#1 plant showed a similar stature as the wild-type R108 plant, and also had a similar shoot and root dry mass as wild-type R108 (Figure 3B,C). The phenotype and dry mass of nodules were also comparable between the *35S:MtBZR1*#1 plant and the wild-type control (Figure 4A–C). 

### 2.5. Transcriptomic Profiles of Nodules in *mtbzr1-1*

**Figure 5:**
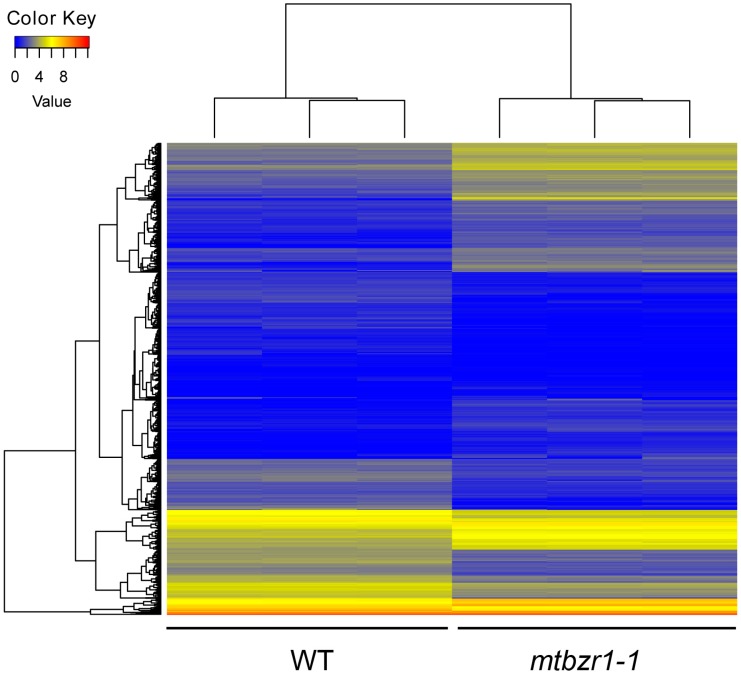
The heatmap of FPKM (fragments per kilobase of transcript per million reads mapped) values of 1319 differential expressed genes in three biological replicates of nodules from *mtbzr1-1* and wild-type plants.

To understand the involvement of *MtBZR1* in nodule development, we performed a transcriptomic assay of *mtbzr1-1.* The nodules were harvested from three biological replicates of *mtbzr1-1* and wild-type plants 21 days after rhizobial inoculation by *S. meliloti* 1021 strain. For each replicate, RNA sequencing generated more than 20 million raw reads, which were filtered and aligned against the annotated reference genome for gene quantification. The correlation efficiency between biological replicates was as high as 99.9%, indicating a good repeatability (Figure S4A, Supplementary Materials). The differentially expressed genes (DEGs) were identified between the *mtbzr1-1* and wild-type sample by DEGseq. Genes with more than two-fold expression changes and a false discovery rate (FDR) smaller than 0.001 were identified as DEGs. A total of 1319 DEGs were found in *mtbzr1-1*, among which 618 were elevated and 701 were suppressed (Figure 5; Figure S4B and Table S2, Supplementary Materials). 

To characterize these differentially expressed genes (DEGs) in *mtbzr1-1*, we carried out enrichment analysis of Gene Ontology (GO) and Kyoto Encyclopedia of Genes and Genomes (KEGG) pathways. Among the most enriched GO terms in *mtbzr1-1′*s DEGs were nodule morphogenesis (GO:0009878), development involved in symbiotic interaction (GO:0044111), multi-organism process (GO:0009877), nodulation (GO:0044403), interspecies interaction between organisms (GO:0044419), symbiosis, encompassing mutualism through parasitism (GO:0044403), and anatomical structure (GO:0048856) (Table 1). These GO categories consisted of the same 43 Nodule Cysteine-Rich (NCR) genes, most of which were under suppression in *mtbzr1-1* compared with wild type (Table 2). The expressions of *MtNCR301* (*Medtr3g015940*), *MtNCR237* (*Medtr7g065025*), *MtNCR327* (*Medtr7g071315*), *MtNCR145* (*Medtr4g060610*), *MtNCR332* (*Medtr4g065455*), and *MtNCR224* (*Medtr6g406350*) were validated by qRT-PCR (Figure S5, Supplementary Materials). The large-scale suppression of these NCRs in *mtbzr1-1* suggests their important roles in nodules, and their deregulation may result in impaired nodule development caused by loss of function of *MtBZR1*.

### 2.6. Differential Expression of Genes Involved in Flavonoid Biosynthesis in *mtbzr1-1*

From the *mtbzr1-1* transcriptome, we identified six significantly enriched secondary metabolite biosynthesis pathways (*p* < 0.01), including isoflavonoid biosynthesis (ko00943), monoterpenoid biosynthesis (ko00902), sesquiterpenoid and triterpenoid biosynthesis (ko00909), flavonoid biosynthesis (ko00941), phenylpropanoid biosynthesis (ko00940), and stilbenoid, diarylheptanoid, and gingerol biosynthesis (ko000945) (Table 3). In the phenylpropanoid biosynthesis pathway, 28 genes were suppressed and 15 were upregulated (Figure S6, Supplementary Materials); in the flavonoid biosynthesis pathway, 16 genes were suppressed and only four genes were upregulated (Figure S7 and Table S3, Supplementary Materials); in the isoflavonoid biosynthesis pathway, 17 genes were suppressed and only three were upregulated (Figure S8 and Table S4, Supplementary Materials). A validation experiment with qRT-PCR showed that two *flavonol synthase* (*FLS*) genes in the flavonoid biosynthesis pathway and four *isoflavone 4′-O-methyltransferase* (*IOMT*) genes in the isoflavonoid biosynthesis pathway were all suppressed in *mtbzr1-1*, consistent with RNA sequencing results (Figure S9, Supplementary Materials). Downregulation of the majority of DEGs involved in the flavonoid and isoflavonoid pathways in *mtbzr1-1* indicates the suppression of their biosynthesis and, thus, possibly reduced production. 

## 3. Discussion

In this study, we characterized *MtBZR1*, a transcriptional regulator of BR signaling in *M. truncatula*, via genetic and molecular approaches. *MtBZR1* expression is elevated in young nodules (seven and 14 dpi) in comparison to roots. Furthermore, the dry mass of shoots and roots was reduced in *mtbzr1-1* compared with wild type. Considering the smaller nodules in *mtbzr1-1* and suppression of genes involved in nodulation, our results demonstrate that *MtBZR1* plays an important role in legume–rhizobia symbiosis.

NCRs play important roles in nodulation in fabaceans [3]. We found that 41 of 43 differential expressed *NCR* genes were under suppression in *mtbzr1-1* compared with wild type. The *NCR* genes are important for the infection, and most of these genes are expressed exclusively in nodules and through the processes of nodule development [17,18]. In our study, the suppression of these *NCR* genes is consistent with nodule dry mass reduction of the *S. meliloti* 1021 strain in *mtbzr1-1*. Within the promoter regions of *NCRs*, conserved motifs such as auxin response factor binding sites were reported [3]. Auxin response factor (ARF) binding sites (TGTCTC) are found to be located within 1000-bp promoter regions after searching for conserved cis-elements and motifs in *NCR* gene promoters [19]. The conserved ARF binding motifs, which are also found in the promoters of *NCR* downregulated in *mtbzr1-1* in this study, suggest the important role of auxin during the symbiosis process between *M. truncatula* and *S. meliloti* 1021. In the pathway of auxin signal transduction, the auxin receptor *TIR1* (*TRANSPORT INHIBITOR RESPONSE 1*) and the series of downstream ARF-related transcription factors are target genes of AtBZR1 in *A. thaliana* [20,21]. Hence, the nodulation-deficient phenotype of *mtbzr1-1* is probably due to MtBZR1′s regulation of the elements downstream of other signaling pathways. 

Flavonoids function as signaling molecules in legume–rhizobia recognition. Specific flavonoids are produced to activate Nod production of rhizobia’s compatible symbionts in conditions with low nitrogen. In accordance with this, the chalcone synthase knockdown in *M. truncatula* disrupts the initiation of nodulation, although the deformation of root hair remains [22]. A diverse group of metabolites derived from the flavonoid biosynthesis pathway accumulate in host roots and nodules. Despite this, only a small number of them clearly demonstrated their role in rhizobial infection. One of these flavonoids is methoxychalcone, which significantly enhances inducing activity on the nodulation genes [8]. It is produced from coumaroyl-CoA by chalcone synthases, whose coding genes are downregulated in the mutant *mtbzr1-1*, and then by chalcone-*O*-methyltransferases. The flavonoids are related to nodulation gene expression in nodules. For example, the infection zones of nodules contain a high level of flavonoids and the nodulation gene expression levels are high [6]. The dysregulation of flavonoid production would impair nodule initiation development in the *mtbzr1-1* mutant. Our data suggest that the suppression of *NCR* genes and flavonoid metabolism probably lead to the impairment of the development of nodules in the cross-talk pathway in *mtbzr1-1*. 

The mutant of the BR 5α-reductase gene in the BR synthesis pathway in pea displayed defective root development and exhibited reduced nodule organogenesis [5]. Furthermore, *mtbzr1-1* developed normal shoots and roots probably due to gene redundancy with its homologs in the genome, similarly to *atbzr1* in *A. thaliana*. Interestingly, *mtbzr1-1* showed defects in nodule development, indicating its different role between shoot development, root development, and nodulation. In the KEGG analysis, the *MtFLSs* were downregulated in *mtbzr1-1*. Their homolog in *A. thaliana*, *At5g05600*, functions as a flavonol synthase and a 2-oxoglutarate/Fe (II)-dependent oxygenase that hydroxylates jasmonic acid (JA) [23]. Consist with this, we found that genes involved in linoleic acid metabolism (ko00591) were enriched with DEGs. Linoleic acids are precursors for JA, which inhibits the calcium spiking induced by Nod factors [23,24]. As an important transcription factor in BR signaling, MtBZR1 might function in hormone cross-talk in nodule development. 

In previous studies, the strain *S. meliloti* 1021 was proven inefficient in nitrogen fixation on A17 and R108 plants [25,26], and its inefficiency might affect the quantitative differences in phenotypes investigated in experiments reported here. However, the *mtbzr1-1* mutant displayed significantly less nodule mass although the numbers of nodules were comparable with the wild-type plants. Despite the possible defective differentiation of bacteroids, the differences in nodule phenotypes between *mtbzr1-1* and wild type were caused by the loss of function of *MtBZR1*. These results indicate that *MtBZR1* is involved in the nodule development after its initiation. Inoculation with an efficient strain, e.g., *S. meliloti* 102F34, would likely result in more pronounced variation in nodule phenotype, which will facilitate screening for new mutants in the future.

## 4. Materials and Methods 

### 4.1. Plant Materials and Growth Conditions

The plant materials used in this work were the *Medicago truncatula* ecotype R108. The *mtbzr1-1* mutant was requested from the Noble Research Institute *Tnt1* Mutant collection [27]. The *mtbzr1-1* mutants were identified using gene-specific and *Tnt1*-specific primers (listed in Table S1, Supplementary Materials). The seeds of wild type, *mtbzr1-1*, and *35S:MtBZR1* were scarified mechanically and vernalized at 4 °C on moist filter paper for one week before transfer to soil. The plants were grown in a growth chamber at 22 °C under a 16-h light/8-h dark cycle with 150 μmol/m^2^/s light intensity and 70–80% relative humidity.

### 4.2. Root Nodule Induction

For rhizobial inoculation, wild-type, *mtbzr1-1*, and *35S:MtBZR1* seedlings were transferred into plastic trays filled with pearlite/sand (in a 3:1 ratio). The plants were grown in the growth chamber and watered every three days with nutrient solution without nitrogen as previously reported [28]. The rhizobium *S. meliloti* 1021 strain was cultured in TY (tryptone, yeast extract, and sodium chloride) medium (supplemented with 10 mg∙mL^−^^1^ tetracycline, 200 mg∙mL^−^^1^ streptomycin, and 6 mmol∙L^−^^1^ calcium chloride) at 28 °C in a shaker to reach an OD_600_ value of 1.0 [28]. Five-day-old seedling of wild type, *mtbzr1-1*, and *35S:MtBZR1* were inoculated with 5 mL of rhizobial suspension diluted to OD_600_ = 0.1. Nodule numbers and dry masses were determined 21 days after inoculation.

### 4.3. Identification of *MtBZR1* Gene and Phylogenetic Analysis

To identify the homologs of *A. thaliana* BZR1 in *M. truncatula*, we used the amino-acid sequences of the two AtBZRs (AtBZR1 and AtBZR2) and four AtBZR1 homologs (AtBEH1 to AtBEH4) from *A. thaliana* for a BLASTP search in the *M. truncatula* (Available online: http://bioinfo3.noble.org/doblast/). A total of seven homologous sequences, Medtr3g111680, Medtr5g019550, Medtr1g021990, Medtr2g075410, Medtr2g075400, Medtr5g026210, and Medtr7g077410, were obtained. To study the phylogenetic relationships, the six amino-acid sequences from *A. thaliana* and seven from *M. truncatula* were aligned in the ClustalW program (Available online: https://www.genome.jp/tools-bin/clustalw). The MYB transcription factors related to AtMYB5, AtTT2, MtMYB5 (Medtr3g083540), and MtMYB14 (Medtr4g125520) acted as the outgroup sequences. Then, the phylogenic tree was constructed by the neighbor-joining, maximum-parsimony, or minimum-likelihood method with 1000 bootstrap replicates in MEGA6 [29].

### 4.4. Plasmid Construction and Plant Transformation 

The coding sequence (CDS) of *MtBZR1* was amplified from a complementary DNA (cDNA) sample prepared from wild-type root RNA with MtBZR1F and MtBZR1R primers (listed in Table S1, Supplementary Materials). The PCR product was purified and cloned into the pENTR/D-TOPO vector (Invitrogen, Carlsbad, CA, USA) and *MtBZR1′*s CDS was confirmed by Sanger sequencing. The resulting pENTR-MtBZR1 plasmid was used to construct *35S:MtBZR1-GFP* for plant transformation by recombination with a destination vector pEarleyGate103 (Invitrogen, Carlsbad, CA, USA) [30]. For *M. truncatula* transformation, the *35S:MtBZR1-GFP* plasmid was introduced into *Agrobacterium* strain EHA105 and used to transform the wild-type plant by the leaf disc method as previously described [31]. The transgenic plants were genotyped with the 35S primer and reverse primer of the gene. The positive ones were accessed for the expression level of *MtBZR1* with its qRT primers for a step further.

The *35S:MtBZR1-GFP* construct or the *35S:GFP* control plasmid were introduced into *Agrobacterium tumefaciens* strain EHA105 and used for an infiltration assay of *Nicotiana benthamiana* leaves as previously reported [32]. Leaves from four-week-old *N. benthamiana* plants were infiltrated with *A. tumefaciens* at an OD_600_ value of 0.4 containing *35S:MtBZR1-GFP* or *35S:GFP*, respectively, and incubated in the dark at 25 °C for 16 h. Leaf discs infiltrated with *A. tumefaciens* were observed using a Zeiss LSM880 confocal microscope at 488-nm wavelength for the GFP fluorescence (Zeiss, Oberkochen, Germany).

### 4.5. RNA Isolation and Quantitative Real-Time PCR (qRT-PCR) 

To analyze the expression level of *MtBZR1* in different organs, samples of root, stem, leaf, flower, and pod were harvested from two-month-old wild-type plants. For time course analysis of *MtBZR1* expression after rhizobial inoculation, nodule samples from wild-type plants were harvested at seven, 14, and 21 days post-inoculation (dpi). To analyze the expression level of *MtBZR1* in *mtbzr1-1* and *35S:MtBZR1* plants, leaves were harvested from one-month-old wild-type, *mtbzr1-1*, and *35S:MtBZR1* plants. For expression analysis of *NCR* genes in *mtbzr1-1* nodules, nodule samples from wild type and *mtbzr1-1* were harvested at 21 dpi. Each biological replicate included 100 mg of nodules collected from 25 plants. To minimize the differences caused by nodule age, we pooled 100 mg of nodules from those plants for RNA extraction. All samples were frozen immediately in liquid nitrogen. RNA was extracted using RNeasy Mini Kit (Qiagen, Beijing, China), and treated with DNase I (Qiagen, Beijing, China). The quantity and quality of extracted RNA were measured by a Nanodrop 2000 Spectrophotometer (NanoDrop Technologies, Waltham, MA, USA). One microgram of total RNA was transcribed into cDNA using ThermoScript RT-PCR system (ThermoFisher, Waltham, MA, USA). After dilution (in a 1:50 ratio), the cDNA samples were used for qRT-PCR on a CFX Connect Real-Time PCR Detection system (Bio-Rad, Hercules, CA, USA) using SYBR Green PCR Master Mix (Roche, Basel, Switzerland) in three biological replicates. An *MtUbiquitin* gene, *Medtr3g110110* [33], was used as an internal control, and relative gene expression was calculated according to the *∆∆C^T^* method [34]. The primer sequences used for qRT-PCR were designed using Primer Express 3.0 software (https://primer-express.updatestar.com/) and are listed in Table S1 (Supplementary Materials).

### 4.6. Transcriptomic Analysis

For the transcriptomic analysis of *mtbzr1-1*, three biological replicates of nodule tissue were harvested from wild-type or *mtbzr1-1* plants 21 days after rhizobial inoculation. RNA samples were sequenced on a BGISEQ-500 platform as eukaryote samples following the manufacturer’s instructions at BGI Genomics Institute (BGI-Shenzhen, Shenzhen, China). About 22 million raw reads were generated for each sample. Adapter sequences, low-quality reads, and reads containing more than 5% unknown nucleotides were first filtered out by trimmonmatic (v0.37) [35]. Clean reads were then aligned to the *M. truncatula* reference transcriptome (version 4.0) using Bowtie [36]. Gene expression (Table S5, Supplementary Materials) was quantified using RSEM (RNA-Seq by Expectation Maximization) [37], and differentially expressed genes with fold change >2 and false discovery rate (FDR) <0.001 were identified using R package DEGseq [38]. The enrichment of Gene Ontology (GO) terms and KEGG pathways was assessed using a hypergeometric test with *p*-values adjusted by the FDR method.

### 4.7. Statistical Analysis

Data processing and statistical analysis were performed using Microsoft^®^ Office Excel 2010 (Available online: http://www.microsoft.com/). A Student’s *t*-test was used to evaluate if the differences were significant in the analysis of gene expression level, nodule numbers, and dry masses between samples.

## 5. Conclusions

In this study, we characterized *MtBZR1*, the homolog of *A. thaliana BZR1* in *M. truncatula*, and found evidence that *MtBZR1* plays an essential role in nodule development. The transcriptomic analysis of the loss-of-function *mtbzr1-1* mutant suggests that MtBZR1 regulates *NCR* and secondary metabolite biosynthesis possibly via hormone signaling cross-talk. This work demonstrates the biological function of *MtBZR1* and a potential role of BR in nodule development, which implies sophisticated hormone cross-talk during nodulation in *M. truncatula.*

## Figures and Tables

**Table 1:** Enriched Gene Ontology (GO) terms in differentially expressed genes (DEGs). ID—identifier.

GO_Term_ID	GO_Term	*q*-Value
GO:0009878	Nodule morphogenesis	2.29 × 10^−10^
GO:0044111	Development involved in symbiotic interaction	2.29 × 10^−10^
GO:0051704	Multi-organism process	2.29 × 10^−10^
GO:0009877	Nodulation	4.77 × 10^−10^
GO:0044419	Interspecies interaction between organisms	4.77 × 10^−10^
GO:0044403	Symbiosis, encompassing mutualism through parasitism	4.80 × 10^−10^
GO:0009653	Anatomical structure morphogenesis	5.30 × 10^−9^
GO:0032502	Developmental process	6.76 × 10^−6^
GO:0048856	Anatomical structure development	1.40 × 10^−5^
GO:0042737	Drug catabolic process	6.72 × 10^−3^
GO:0005975	Carbohydrate metabolic process	7.35 × 10^−3^

**Table 2:** The expression of genes involved in GO:0009878. FDR—false discovery rate.

Gene	Annotation	log2FC	FDR
Medtr6g462060	NCR secreted peptide	−1.03955	9.79 × 10^−^^86^
Medtr4g065410	Late nodulin	−1.30701	1.6 × 10^−10^^6^
Medtr4g065455	NCR secreted peptide	−1.1222	3.27 × 10^−^^24^
Medtr0337s0030	NCR secreted peptide	−1.09355	4.47 × 10^−^^64^
Medtr6g406350	NCR secreted peptide	−1.66085	1.45 × 10^−10^
Medtr1g037670	NCR secreted peptide	−1.01068	2.46 × 10^−^^36^
Medtr4g060370	Late nodulin	−1.03975	1 × 10^−1^^36^
Medtr4g063740	NCR secreted peptide	−1.30404	4.43 × 10^−^^2^^0^
Medtr5g063560	Hypothetical protein	−1.29477	1.21 × 10^−1^^1^
Medtr0050s0100	NCR secreted peptide	−1.10619	2.68 × 10^−^^36^
Medtr6g445080	NCR secreted peptide	−1.06689	0
Medtr7g008130	NCR secreted peptide	−1.28862	1.51 × 10^−^^56^
Medtr7g056047	NCR secreted peptide	−1.04601	1.2 × 10^−1^^1^
Medtr7g045410	NCR secreted peptide	−1.16667	5.16 × 10^−^^8^
Medtr5g459510	NCR secreted peptide	−1.31551	1.5 × 10^−1^^11^
Medtr6g445020	NCR secreted peptide	−1.34506	3.19 × 10^−^^78^
Medtr6g461840	NCR secreted peptide	−1.00809	1.7 × 10^−10^^8^
Medtr6g044700	Unknown	−1.26281	1.1 × 10^−1^^13^
Medtr1g028980	NCR secreted peptide	−1.0595	4.01 × 10^−^^37^
Medtr7g071315	NCR secreted peptide	−1.13168	1.4 × 10^−1^^21^
Medtr3g071360	NCR secreted peptide	−1.08081	3.83 × 10^−^^74^
Medtr2g066255	Late nodulin	−1.06841	1.7 × 10^−^^5^^0^
Medtr7g045520	NCR secreted peptide	−1.08055	0
Medtr7g406940	Late nodulin	−1.24693	1.01 × 10^−^^28^
Medtr3g063450	Late nodulin	−1.10333	1.25 × 10^−^^48^
Medtr2g072970	Late nodulin	−1.03982	1.13 × 10^−^^9^
Medtr0330s0030	NCR secreted peptide	−1.14577	1.01 × 10^−^^4^
Medtr7g015880	NCR secreted peptide	−1.21986	1.48 × 10^−^^32^
Medtr2g450150	NCR secreted peptide	−1.0796	2.94 × 10^−^^24^
Medtr2g044310	NCR secreted peptide	−1.08089	3.88 × 10^−^^4^
Medtr7g102806	NCR secreted peptide	−1.36812	1.68 × 10^−^^33^
Medtr4g017790	NCR secreted peptide	−1.28877	1.1 × 10^−1^^4^
Medtr5g059420	NCR secreted peptide	1.234336	1.19 × 10^−1^^1^
Medtr7g445930	NCR secreted peptide	−1.09898	9.81 × 10^−^^5^
Medtr7g008020	NCR secreted peptide	−1.28923	4.52 × 10^−^^6^
Medtr5g048335	NCR secreted peptide	−1.22601	2.84 × 10^−^^77^
Medtr2g044330	NCR secreted peptide	−1.18661	0
Medtr7g071585	Late nodulin	−1.05481	2.36 × 10^−1^^2^
Medtr5g044135	Late nodulin	−1.49748	4.23 × 10^−^^66^
Medtr2g022740	NCR secreted peptide	−1.08612	5.77 × 10^−^^75^
Medtr7g065025	NCR secreted peptide	−1.00326	1.49 × 10^−^^98^
Medtr1g046020	NCR secreted peptide	−1.03343	3.44 × 10^−^^52^
Medtr6g060320	NCR secreted peptide	−1.39606	1.5 × 10^−^^5^
Medtr3g015940	NCR secreted peptide	−1.32883	2.4 × 10^−^^63^
Medtr7g045910	NCR secreted peptide	−1.30758	3.5 × 10^−^^21^
Medtr7g055933	NCR secreted peptide	−1.08339	3.44 × 10^−^^4^
Medtr6g027155	NCR secreted peptide	−1.13114	6.91 × 10^−^^69^
Medtr6g478110	Kunitz-type trypsin inhibitor/miraculin	−1.15167	3.82 × 10^−^^47^
Medtr6g038620	NCR secreted peptide	−1.25415	1.84 × 10^−1^^6^
Medtr7g064970	Late nodulin	1.213179	1.85 × 10^−^^6^
Medtr5g058510	NCR secreted peptide	−1.10207	6.12 × 10^−^^7^^0^
Medtr5g072205	NCR secreted peptide	1.301282	7.7 × 10^−^^4^
Medtr3g071330	NCR secreted peptide	−1.05226	1.49 × 10^−^^54^
Medtr7g033855	Low-molecular-weight cysteine-rich (LCR) protein	−1.05107	4.07 × 10^−^^4^
Medtr4g060610	NCR secreted peptide	−1.06185	1.25 × 10^−^^25^
Medtr6g060370	NCR secreted peptide	−5.53715	1.77 × 10^−^^22^

**Table 3:** Enriched KEGG pathways in DEGs.

KEGG_Term_ID	KEGG_Term	*q*-Value
ko00943	Isoflavonoid biosynthesis	5.91 × 10^−7^
ko00902	Monoterpenoid biosynthesis	9.52 × 10^−7^
ko00909	Sesquiterpenoid and triterpenoid biosynthesis	8.49 × 10^−5^
ko00941	Flavonoid biosynthesis	8.84 × 10^−4^
ko00940	Phenylpropanoid biosynthesis	4.34 × 10^−3^
ko04712	Circadian rhythm-plant	7.15 × 10^−3^
ko00620	Pyruvate metabolism	1.66 × 10^−2^
ko00591	Linoleic acid metabolism	7.93 × 10^−2^
ko00010	Glycolysis/gluconeogenesis	9.55 × 10^−2^
ko00945	Stilbenoid, diarylheptanoid, and gingerol biosynthesis	9.55 × 10^−2^

## Supplementary Materials

Supplementary materials can be found at https://www.mdpi.com/1422-0067/20/12/2941/s1. Figure S1. The maximum-parsimony and maximum-likelihood trees of BZR1-like proteins in *A. thaliana* and *M. truncatula*. (A) The maximum-parsimony tree of BZR1-like proteins. (B) The maximum-likelihood tree of BZR1-like proteins. Figure S2. Multiple alignment of MtBZR1, BZR1, and BZR1-like proteins from *A. thaliana* and *M. truncatula*. The BZR domain is indicated by a red line. Identical and similar amino acids are indicated as asterisks and colons. Figure S3. The expression of *MtBZR1* in *35S:MtBZR1* transgenic lines. (A) The expression of *MtBZR1* in *35S:MtBZR1* transgenic lines. (B) The leaves from WT and *35S:MtBZR*#1 transgenic line. (C) GFP signal was detected in tobacco leaf disc two days after infiltration with *Agrobacterium* carrying a *35S:MtBZR1-GFP* construct. The *35S:GFP* construct was used as a negative control. Scale bar = 0.5 cm in (B) and 100 µm in (C). Figure S4. Repeatability and differential analysis of RNA-sequencing data. (A) The heatmap of Pearson’s coefficient between sequencing samples. (B) The volcano plot of differential analysis between *mtbzr1-1* and wild-type plants. The induced and suppressed genes are indicated as red and green points, respectively. Figure S5. The expressions of *MtNCRs*, *MtNCR301*, *MtNCR237*, *MtNCR327*, *MtNCR145*, *MtNCR332*, and *MtNCR224* in *mtbzr1-1* and wild-type plants. *MtUbiquitin* was used as an internal control. Bars represent standard errors from three biological replicates. Figure S6. The phenylpropanoid biosynthesis pathway with induced and suppressed genes indicated in red and green frames, respectively. Figure S7. The flavonoid biosynthesis pathway with induced and suppressed genes indicated in red and green frames, respectively. Figure S8. The isoflavonoid biosynthesis pathway with induced and suppressed genes indicated in red and green frames, respectively. Figure S9. The expressions of *MtFLSs* and *MtIOMTs* in *mtbzr1-1* and wild-type plants. *MtUbiquitin* was used as an internal control. Bars represent standard errors from three biological replicates. Table S1. Primers used in this study. Table S2. The 1319 differentially expressed genes in *mtbzr1-1*. Table S3. The expression of genes involved in the flavonoid biosynthesis pathway. Table S4. The expression of genes involved in the isoflavonoid biosynthesis pathway. Table S5. The expression of all genes in WT and *mtbzr1-1*.

## Abbreviations

ARF	AUXIN RESPONSE FACTOR
BES1	BRASSINOSTEROID INSENSITIVE 1-EMS SUPPRESSOR 1
BR	brassinosteroid
BZR	BRASSINAZOLE RESISTANT 1
CDS	coding sequence
DEG	differentially expressed gene
DPI	days post inoculation
FDR	false discovery rate
FLS	flavonol synthase
FPKM	fragments per kilobase of transcript per million reads mapped
GFP	green fluorescence protein
GO	gene ontology
IOMT	isoflavone *O*-methyltransferase
JA	jasmonic acid
KEGG	Kyoto encyclopedia of genes and genomes
LCR	low-molecular-weight cysteine-rich
NCR	nodule-specific cysteine-rich
qRT-PCR	quantitative real-time PCR
RSEM	RNA-seq by expectation maximization
TIR1	TRANSPORT INHIBITOR RESPONSE 1
TT2	TRANSPARENT TESTA 2
TY	tryptone, yeast extract, and sodium chloride
WT	wild type
