# Supplementary material for: MtBZR1 Plays an Important Role in Nodule Development in Medicago truncatula
Source: Int J Mol Sci. 2019 Jun 16;20(12):2941. doi: 10.3390/ijms20122941 (PMC6627309; doi:10.3390/ijms20122941)
Supplement: Supplementary file 1 [file ijms-20-02941-s001.zip › ijms-510599 suppl.docx]

*MtBZR1* Plays an Important Role in Nodule Development in *Medicago truncatula*





Figure S1. The maximum-parsimony and maximum-likelihood trees of BZR1-like proteins in *A. thaliana* and *M. truncatula*. (A) The maximum-parsimony tree of BZR1-like proteins. (B) The maximum-likelihood tree of BZR1-like proteins.


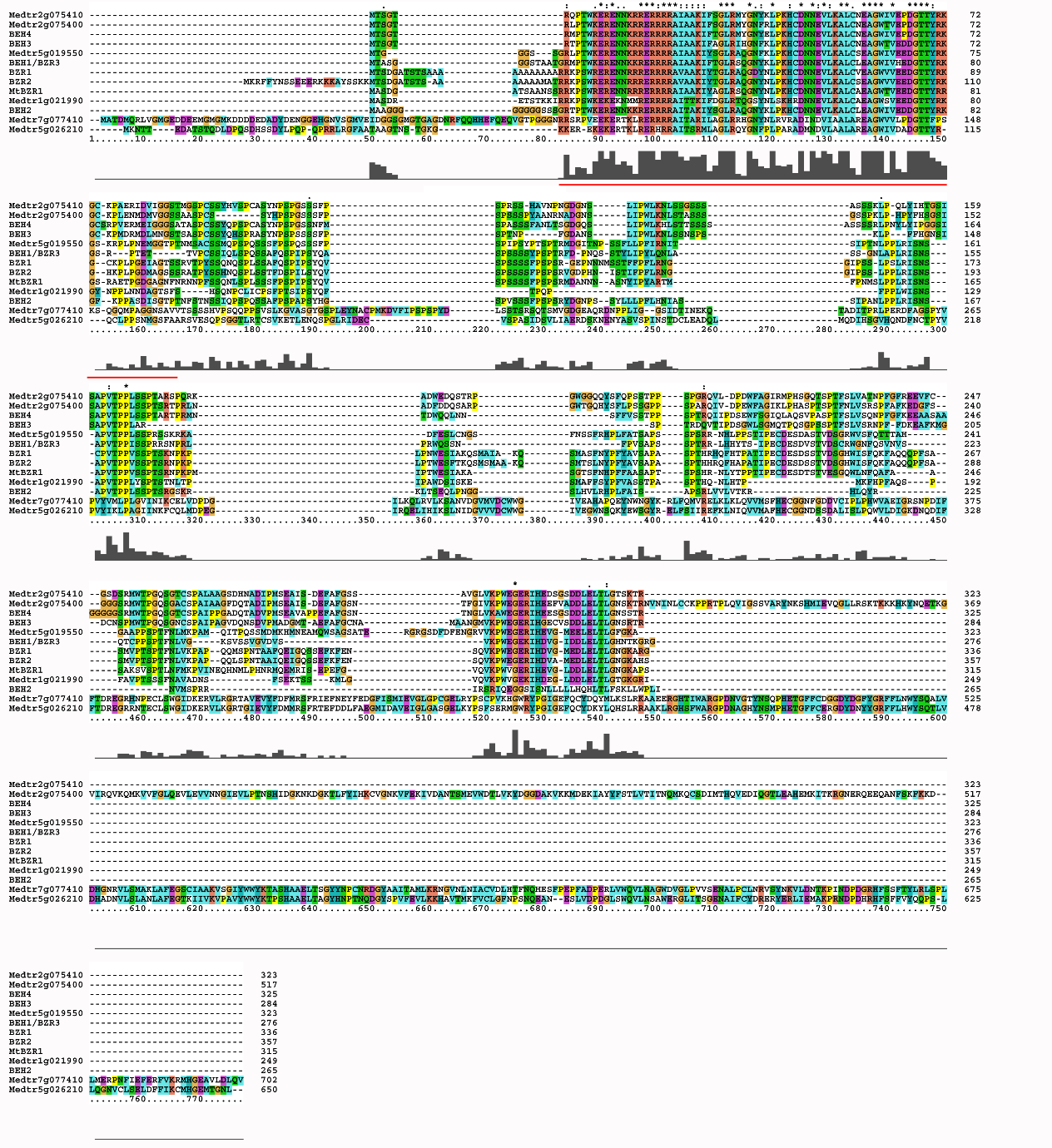


Figure S2. Multiple alignment of MtBZR1, BZR1, and BZR1-like proteins from *A. thaliana* and *M. truncatula*. The BZR domain is indicated by a red line. Identical and similar amino acids are indicated as asterisks and colons.


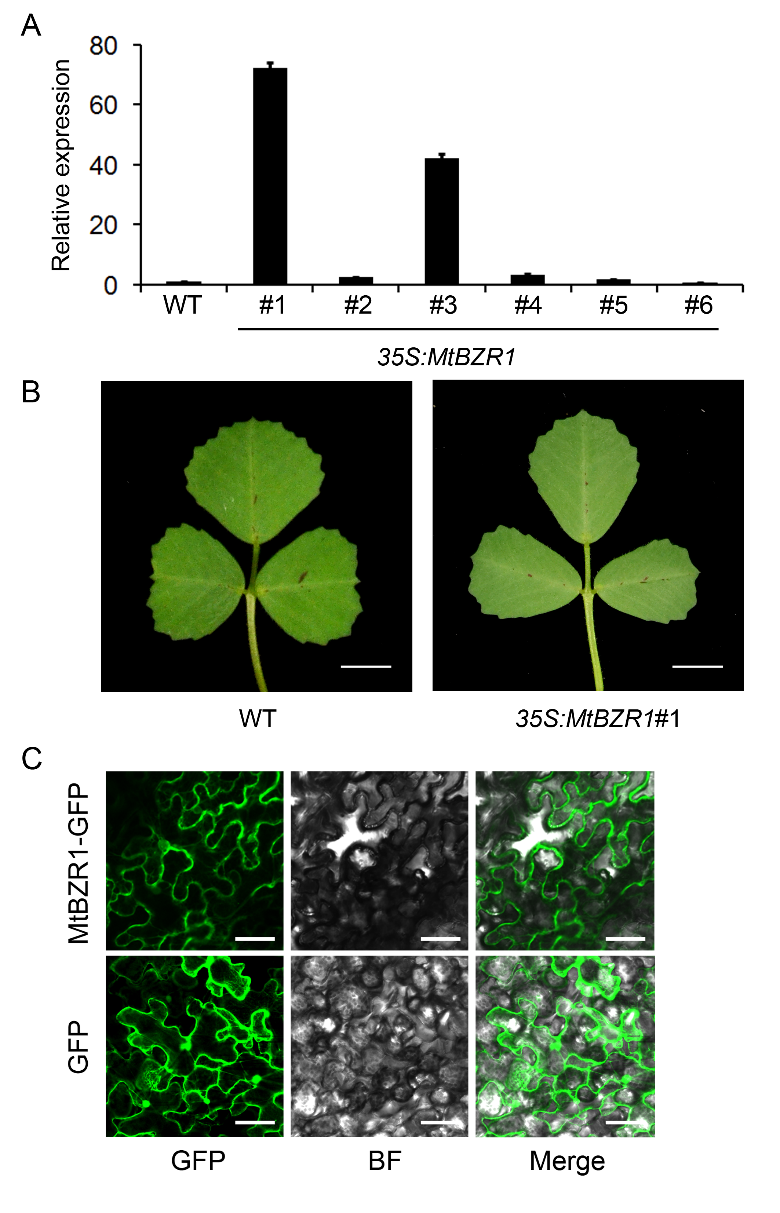


Figure S3. The expression of *MtBZR1* in *35S:MtBZR1* transgenic lines. (A) The expression of *MtBZR1* in *35S:MtBZR1* transgenic lines. (B) The leaves from WT and *35S:MtBZR*#1 transgenic line. (C) GFP signal was detected in tobacco leaf disc two days after infiltration with *Agrobacterium* carrying a *35S:MtBZR1-GFP* construct. The *35S:GFP* construct was used as a negative control. Scale bar = 0.5 cm in (B) and 100 µm in (C).


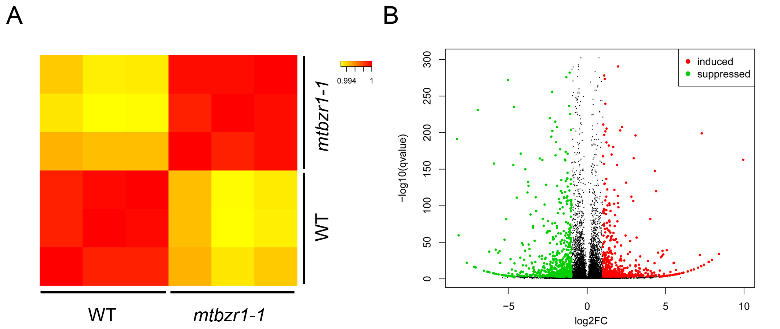


Figure S4. Repeatability and differential analysis of RNA-sequencing data. (A) The heatmap of Pearson’s coefficient between sequencing samples. (B) The volcano plot of differential analysis between *mtbzr1-1* and wild-type plants. The induced and suppressed genes are indicated as red and green points, respectively.


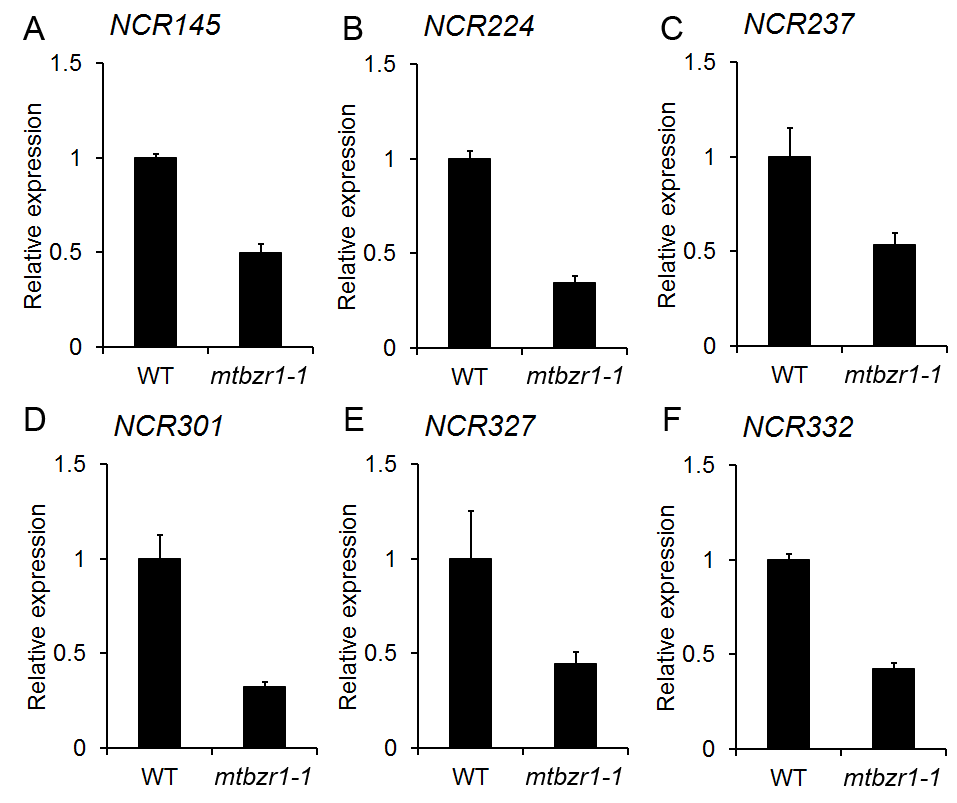


Figure S5. The expressions of *MtNCRs*, *MtNCR301*, *MtNCR237*, *MtNCR327*, *MtNCR145*, *MtNCR332*, and *MtNCR224* in *mtbzr1-1* and wild-type plants. *MtUbiquitin* was used as an internal control. Bars represent standard errors from three biological replicates.


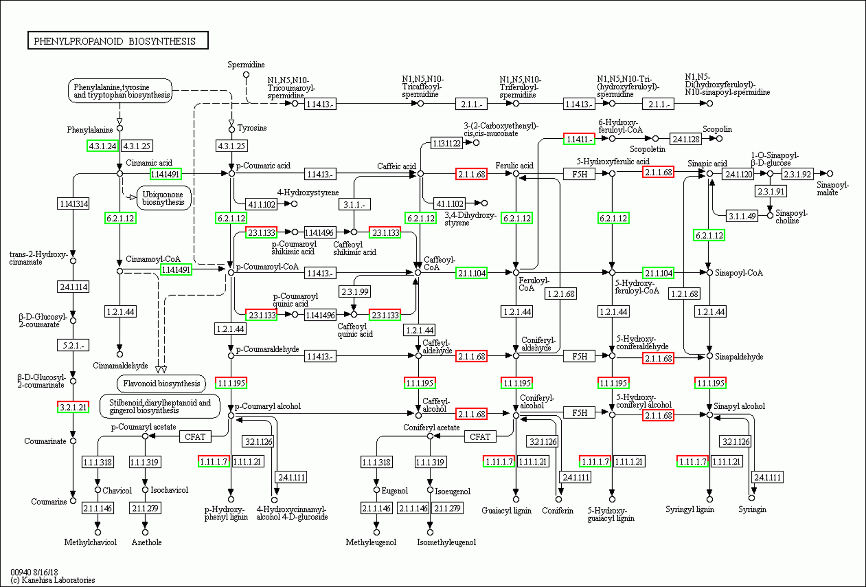


Figure S6. The phenylpropanoid biosynthesis pathway with induced and suppressed genes indicated in red and green frames, respectively.


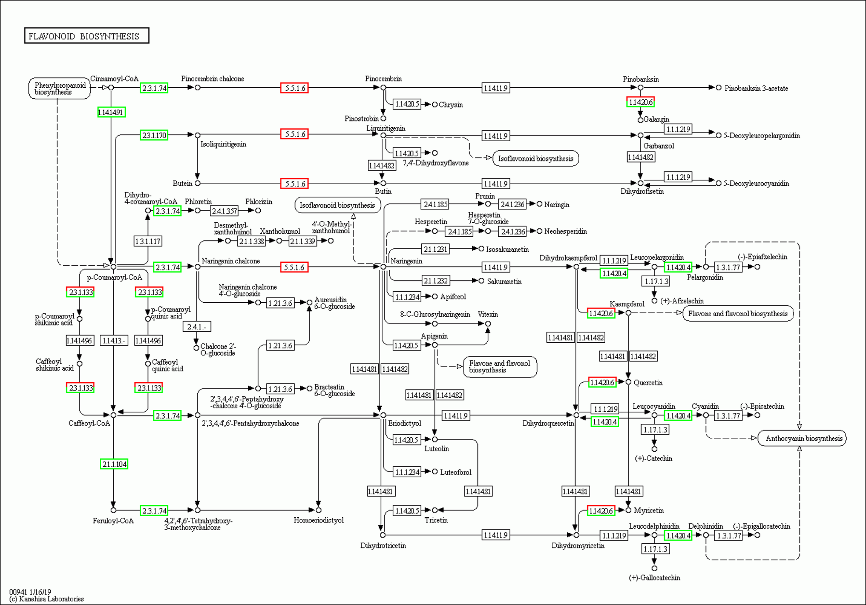


Figure S7. The flavonoid biosynthesis pathway with induced and suppressed genes indicated in red and green frames, respectively.


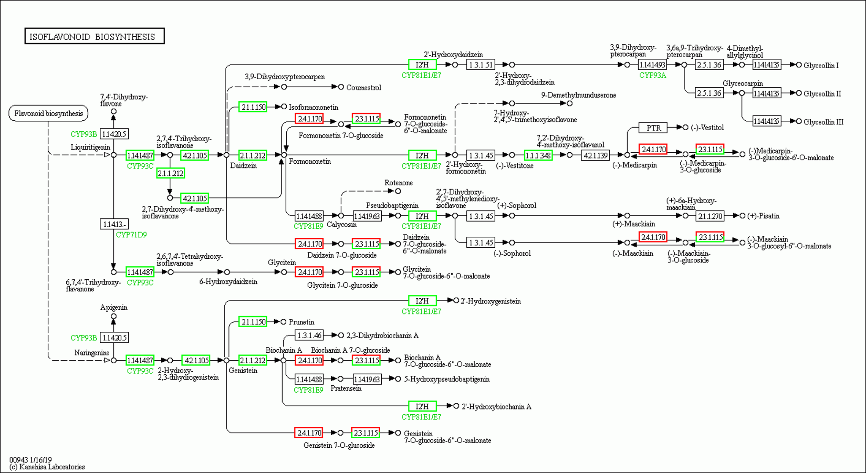


Figure S8. The isoflavonoid biosynthesis pathway with induced and suppressed genes indicated in red and green frames, respectively.


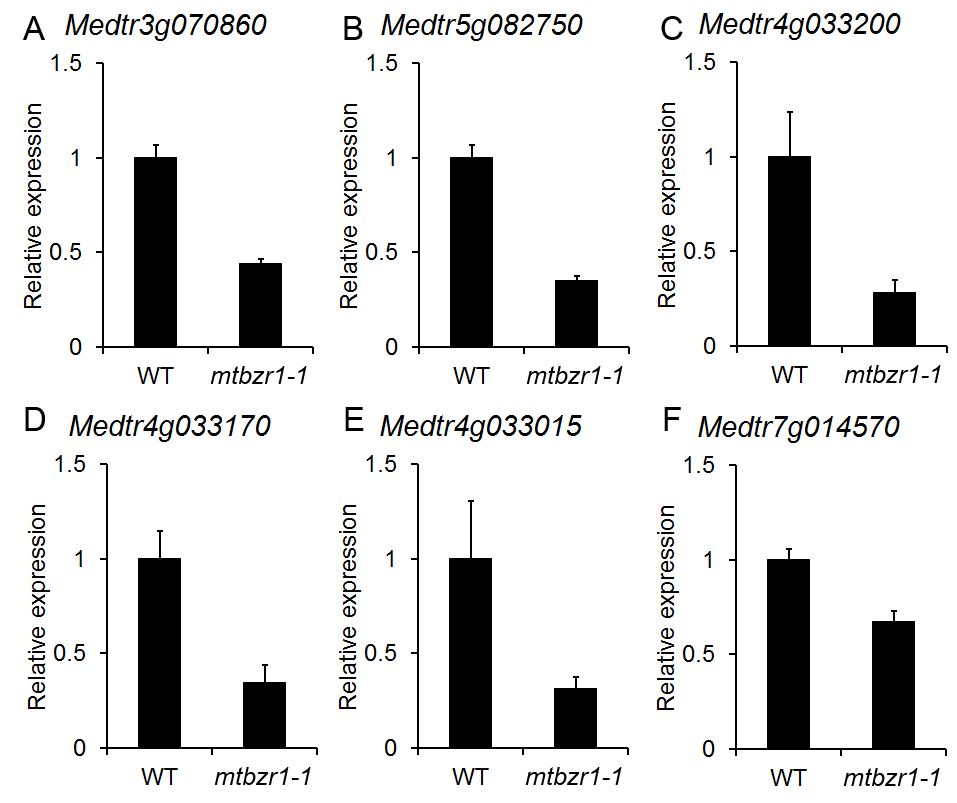


Figure S9. The expressions of *MtFLSs* and *MtIOMTs* in *mtbzr1-1* and wild-type plants. *MtUbiquitin* was used as an internal control. Bars represent standard errors from three biological replicates.
